# Detection of α-Galactosidase A Reaction in Samples Extracted from Dried Blood Spots Using Ion-Sensitive Field Effect Transistors

**DOI:** 10.3390/s24113681

**Published:** 2024-06-06

**Authors:** Alexander Kuznetsov, Andrey Sheshil, Eugene Smolin, Vitaliy Grudtsov, Dmitriy Ryazantsev, Mark Shustinskiy, Tatiana Tikhonova, Irakli Kitiashvili, Valerii Vechorko, Natalia Komarova

**Affiliations:** 1 Institute of Nanotechnology of Microelectronics of the Russian Academy of Sciences, 32A Leninsky Prospekt, Moscow 119334, Russia; 2 Municipal Clinical Hospital No. 15 named after O.M. Filatov, 23 Veshnyakovskaya St., Moscow 111539, Russia

**Keywords:** newborn screening, Fabry disease, ion-sensitive field effect transistor, α-galactosidase A, detection

## Abstract

Fabry disease is a lysosomal storage disorder caused by a significant decrease in the activity or absence of the enzyme α-galactosidase A. The diagnostics of Fabry disease during newborn screening are reasonable, due to the availability of enzyme replacement therapy. This paper presents an electrochemical method using complementary metal-oxide semiconductor (CMOS)-compatible ion-sensitive field effect transistors (ISFETs) with hafnium oxide-sensitive surfaces for the detection of α-galactosidase A activity in dried blood spot extracts. The capability of ISFETs to detect the reaction catalyzed by α-galactosidase A was demonstrated. The buffer composition was optimized to provide suitable conditions for both enzyme and ISFET performance. The use of ISFET structures as sensor elements allowed for the label-free detection of enzymatic reactions with melibiose, a natural substrate of α-galactosidase A, instead of a synthetic fluorogenic one. ISFET chips were packaged with printed circuit boards and microfluidic reaction chambers to enable long-term signal measurement using a custom device. The packaged sensors were demonstrated to discriminate between normal and inhibited GLA activity in dried blood spots extracts. The described method offers a promising solution for increasing the widespread distribution of newborn screening of Fabry disease.

## 1. Introduction

Newborn screening (NBS) from dried blood spots (DBSs) is a highly effective secondary prevention measure that has been established for many years. Since its beginning in the United States in the mid-1960s, NBS has been an extremely successful public health program, saving thousands of lives [1]. Recently, pilot NBS programs for Fabry disease (FD) have been implemented worldwide to explore the potential inclusion of this disease in national programs in Europe [2,3], Japan [4], China [5], and the USA [6]. These results have shown that FD is surprisingly more common than previously considered [7]. FD is associated with lysosomal glycohydrolase α-galactosidase A (GLA) deficiency, which causes premature death due to cardiovascular disease, kidney failure, and strokes, if not treated from birth with the recommended enzyme replacement therapy [8,9]. The methods used for pilot NBS programs for FD are based on fluorometry, digital microfluidics, tandem mass spectrometry, and immune quantification and are discussed in detail in the review by Gragnianiello and colleagues [7]. However, despite the variety of methods currently available for the diagnosis of this disease, for the widespread dissemination of newborn screening, it is necessary to improve the cost of analysis, accuracy, parallelism, and extraction of information from a limited amount of biological material. Modern complementary metal-oxide semiconductor (CMOS) technologies make it possible to create semiconductor sensor arrays which meet the challenges faced by diagnostics aimed at newborn screening. Ion-sensitive field effect transistors (ISFETs) are a promising type of transducer that can be monolithically integrated with signal processing circuits. It allows the registration of direct molecule interactions and is a popular part used in biosensors for the determination of biomarkers [10,11,12], low molecular weight compounds [13,14], DNA–DNA interactions [15,16], or cell viability [17,18]. In addition, ISFETs are solid-state pH sensors that are able to detect local pH changes [19]. In this paper, we show that ISFET sensors formed within the framework of post-fab processing can be used to diagnose the activity of the GLA enzyme and in the future, can become the basis for the diagnosis of Fabry disease in newborns.

## 2. Materials and Methods

### 2.1. Materials

Recombined human α-Galactosidase A (GLA) protein was obtained from Bio-Techne Corp., Minneapolis, MN, USA. 4-methylumbelliferyl-α-d-galactopyranoside and deoxygalactonojirimycin were purchased from Cayman Chemical Company, Ann Arbor, MI, USA. Melibiose was purchased from Macklin Biochemical Technology Co., Ltd., Shanghai, China. Citric acid, sodium citrate, sodium phosphate dibasic dihydrate, tris base, and glycine were obtained from Sigma, Roedermark, Germany. ISFETs were manufactured according to the standard CMOS process. The extended gate with HfO_2_ was formed by a post-processing cycle, according to [20].

### 2.2. Device and Microfluidic System Formation

ISFETs were wired in printed circuit boards and packaged in microfluidic chambers that were formed using 3D printing. Au wire was used as the reference electrode. Surface potential measurements were performed with a portable homemade device. ISFET measurements were carried out by setting the reference voltage and the operating point in the weak inversion mode using the portable device. The device was programmed with the ability to measure ISFET I–V characteristics and to determine the subthreshold swing. Using the transimpedance amplifier circuit, the ISFET drain current was converted to output voltage. The relative change of the floating gate potential was determined from the output voltage, taking into account the ISFET subthreshold swing and known circuit parameters. The visualization and processing of the results was performed using software developed using Python 3.12 to support a graphical user interface, and was installed on a laptop connected via USB to the portable device.

### 2.3. Enzyme Activity Measurements

#### 2.3.1. Fluorescent Measurements

GLA activity was measured using 4-methylumbelliferyl-α-d-galactopyranoside (4-MU-α-Gal) as a substrate [21]. The reaction was carried out in black 96-well microplates. Each well contained 100 µL of reaction mixture with 1.5 mM 4-MU-α-Gal and 1.2 × 10^−7^ M GLA. The enzyme activity was assayed in citrate–phosphate and citrate–citrate buffers with different pH and molarity. After 30–45 min of reaction, 200 µL of stop-buffer (0.2 M tris-glycine, pH 10.5) was added to the wells, and the end-point fluorescent signal of the reaction product, 4-methylumbelliferone (ex/em 350/460 nm), was detected using the Infinite M200 PRO modular plate reader (‘Tecan’, Männedorf, Switzerland).

#### 2.3.2. ISFET Measurements Using a Semiconductor Parameter Analyzer

The optimization of buffer composition and studies of GLA reaction with melibiose on ISFETs were performed using an Agilent B1500A semiconductor device parameter analyzer (Agilent Technologies, Inc., Santa Clara, CA, USA) and an H8 probe station (Semishare Co., Ltd., Shenzhen, China). A Ag/AgCl reference electrode was used to make contact with the buffer solution, which was contained in a well-like structure formed on the surface of a chip using the direct ink writing method. Using the subthreshold swing value obtained by measuring transfer I–V characteristics, time-dependent changes in the current I_D_ (V_G_ = const, V_DS_ = 0.1 V) were converted into surface potential (Δφs), as described in earlier work [22], and were used as analytical signals. Prior to the measurements, the structures were conditioned in buffer for stabilization and drift reduction.

#### 2.3.3. Measurements of the Samples Extracted from Dry Blood Spots

Blood samples were collected from adult patients not diagnosed with FD. Informed consent was obtained.

To prepare GLA extracts, 400 µL of 20 mM citrate–phosphate buffer pH 4.5 was added to 2 pcs of 6 mm DBS punch. The extraction was performed at 37 °C and 1200 rpm for 1 h.

To detect GLA activity in DBS extracts with fluorescent measurements, 70 µL of the extract was used to obtain 260 µL of reaction mixture with 135 µM 4-MU-α-Gal concentration. In the control experiment, 115 nM of GLA inhibitor deoxygalactonojirimycin [23] (DGJ) was added to the reaction mixture. After 20 h of incubation at 37 °C and 1200 rpm, the reaction was stopped by the addition of 50 µL of 1.32M ethylenediamine solution, and the fluorescence of 4-methylumbelliferone was detected using the DeNovix DS-11 FX fluorometer (DeNovix Inc., Wilmington, DE, USA).

To detect GLA activity with ISFETs, 38 µL of extract sample was mixed with 17 µL of reaction buffer and 2 µL of 500 mM melibiose, and was added to the device. Alternatively, 38 µL of extracts were mixed with 17 µL of 1 µM DGJ and 2 µL of 500 mM melibiose.

## 3. Results and Discussion

### 3.1. ISFET Fabrication

**Figure 1:**
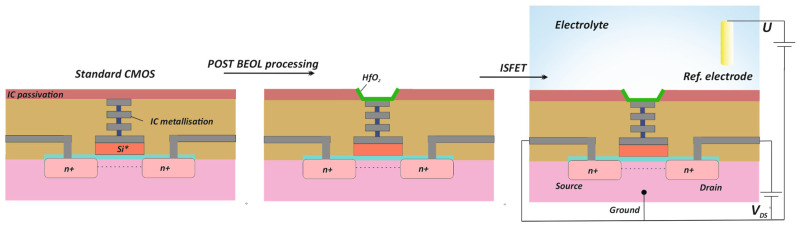
Technological route of ISFET fabrication with post-BEOL processing. Si*—polycrystalline silicon.

For the study of GLA reaction, ISFETs compatible with the standard routes of commercial CMOS factories have been used. The process of structure formation illustrated in Figure 1 implies the post-BEOL (back end of line) treatment of wafers to form the ISFETs. In wafers made using the standard CMOS process, the passivation is exposed to the aluminum pads of the MOS transistors connected to the gate. An ISFET is then formed based on the MOS transistor, by forming a hafnium oxide layer on top of the aluminum pad using atomic layer deposition, which provides sensitivity close to the Nernst limit ≈ 59 mV/pH [24]. The developed manufacturing route, in conjunction with the ISFET design, makes it possible to minimize the known non-ideality of ISFETs [25], i.e., the effect of capacitive attenuation, trapped charge, and drift. The experimental comparison of this technological route with unmodified CMOS was previously performed [20]. Since it was assumed that the measurements would be carried out in the subthreshold transistor mode, the structures were designed to operate in weak inversion mode. The ISFET was designed to meet the requirement of maintaining the subthreshold slope at a value similar to that of MOSFET. For this purpose, the calculation given in [20] was performed for a hafnium oxide film obtained using the ALD method, with a transistor with a size of 500/4 (W/L) and a thickness of the gate oxide of 32.5 nm. The final structure contained a sensitive area of 110 × 110 µm with an opening area of 100 × 100 µm, on top of which, 35 nm of hafnium oxide was deposited (Figure S1). For these structures, the average subthreshold swing was 76 ± 0.5 mV/dec, and the pH dependence of 56 mV/pH using a Ag/AgCl reference electrode and 0.1 M citrate–phosphate buffer was obtained. This value is in good agreement with data found in the literature for ISFETs with a sensitive surface based on hafnium oxide [26,27].

### 3.2. Optimization of Buffer Composition

Human α-galactosidase A (α-d-galactoside galactohydrolase; EC 3.2.1.22; α-Gal A) is an enzyme that cleaves the terminal α-d-galactosyl moieties from glycolipids and glycoproteins [28]. ISFETs are well-known solid-state pH sensors that are capable of detecting pH changes during enzymatic reactions [29]. The buffer composition strongly influences both the enzyme activity and the ISFET sensitivity. An enzyme displays the highest activity in its appropriate buffer, while the ISFET performance for pH changing increases with the decreasing ionic strength of the gate solution [30]. Therefore, the selection of the optimal buffer composition ensuring sufficient GLA activity and ISFET sensitivity is an essential step for biosensor development.

**Figure 2:**
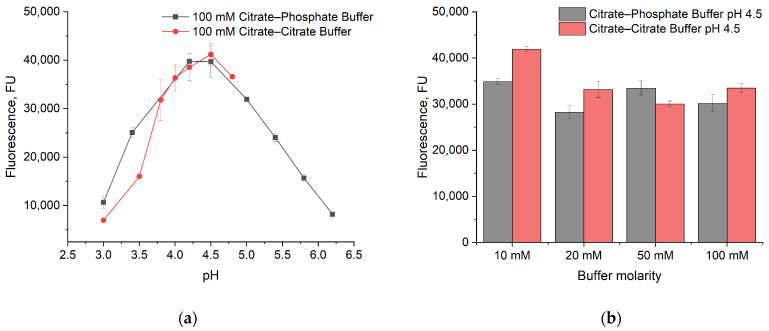
(**a**) Dependence of GLA activity on buffer pH; (**b**) dependence of GLA activity on buffer molarity.

The dependence of GLA activity on pH was measured in 100 mM citrate–phosphate and 100 mM citrate–citrate buffers (Figure 2a) using a fluorescent assay. These buffers are usually used for the measurement of GLA activity [21,31,32]. In both buffers, the highest enzyme activity was observed at pH 4.5 (Figure 2a). The maximum signal values were nearly equal, regardless of the buffer type.

The dependence on the buffer molarity was studied for both buffers at a fixed pH of 4.5 (Figure 2b) using fluorescent detection. No obvious dependence was observed for the citrate–phosphate buffer. In the citrate–citrate buffer, enzyme activity increased with decreasing buffer molarity.

To test the response of the transistor to an enzymatic reaction, the catalytic hydrolysis of 4-MU-α-Gal was initiated in a gate solution of ISFETs, and the surface potential in the subthreshold mode of the transistor was measured. Experiments with variable order of the addition of reagents showed that a noticeable change in surface potential occurs only in the joint presence of all components in solution (Figure S2). Since the enzyme addition step caused a high signal distortion, in all subsequent experiments, the reaction was initialized by the addition of the substrate.

A comparison of the two buffer systems on ISFETs showed that for the citrate–citrate buffer solution, the baseline noise and drift were observed, leading to non-reproducible data (Figure S3).

**Figure 3:**
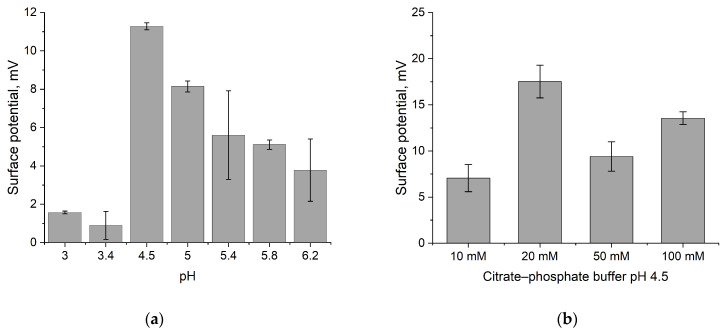
(**a**) Dependence of ISFET response to GLA reaction in 100 mM citrate–phosphate buffer with different pH values; (**b**) dependence of ISFET response to GLA reaction in citrate–phosphate buffer (pH 4.5) with different molarities.

The dependence of the sensor response to the enzymatic reaction was studied at different pH values of the citrate–phosphate buffer (Figure 3a). As in the case of the fluorescent study, the greatest sensor response was obtained at pH values corresponding to the highest enzyme activity, while reducing the molarity from 50 mM to 20 mM allowed a 1.6-fold increase in the enzyme response under the same experimental conditions (Figure 3b). A further decrease in molarity led to a background response to the addition of reagents and an increase in ISFET drift over time, which greatly complicated the subsequent interpretation of the results. Thus, the optimal conditions for detecting the hydrolysis reaction catalyzed by GLA were established as a 20 mM citrate–phosphate buffer with a pH of 4.5.

### 3.3. GLA Reaction with Native Substrate

**Figure 4:**
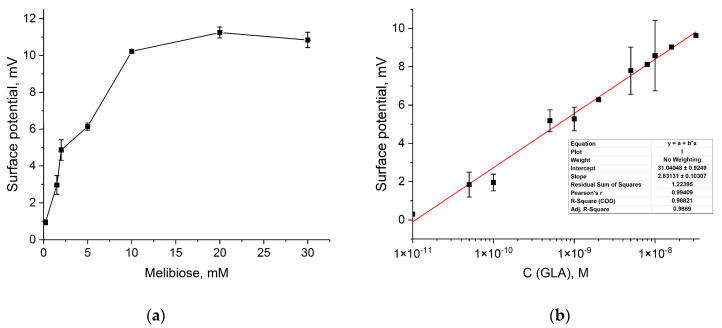
(**a**) Dependence of ISFET response to GLA reaction with different melibiose concentrations; (**b**) calibration curve for GLA detection using ISFETs (20 mM citrate–phosphate buffer, pH 4.5, 30 mM melibiose). The limit of detection was calculated to be 9.56 × 10^−11^ M using 3.3 standard deviations.

The undoubted advantage of ISFETs is the possibility of the direct detection of the enzymatic reaction without the use of fluorescent or electroactive molecules. The possibility of detecting the reaction catalyzed by GLA using a native substrate has been studied with melibiose. For this purpose, the GLA reaction response was studied in a melibiose concentration range of 0.2–30 mM under established conditions. Similarly to the reaction with 4-MU-α-Gal, the relative change in surface potential increased with the increase in the concentration of melibiose in the gate solution. At the same time, the signal reached saturation at a melibiose concentration of 20 mM (Figure 4a).

The study of the reaction showed that there is no substrate inhibition for melibiose, which means that melibiose in saturation concentration can be used to detect the reaction without limitations. Finally, for the melibiose substrate at saturation concentration and under optimal conditions, the response of the ISFET to the GLA reaction was studied for different enzyme concentrations ranging from 1 × 10^−11^ M to 3.2 × 10^−8^ M (Figure 4b). Experiments have shown that the relative change in surface potential depends on the concentration of the enzyme, and the reaction was reliably detected at the concentration of the enzyme, 1 × 10^−10^ M, with an exposure time of 15 min. At the same time, under similar conditions in the fluorescent assay, the concentration became detectable at 1 × 10^−10^ M, with an exposure time of 45 min. In addition, the observed difference in the rate of signal accumulation may be due to a decrease in the volume in which the measurement is performed; a similar acceleration effect for the GLA-catalyzed reaction is observed when transferring the fluorescent assay to the digital microfluidic format [33].

Thus, it can be concluded from preliminary experiments on a chip that the accumulation of the signal due to GLA reaction on the ISFET is faster, which should allow for analysis using known benefits of lab-on-a-chip approaches, whereby the smaller the consumption of reagent, the shorter the time of analysis and additional automatization [34].

### 3.4. Sensor Characterization with Au Pseudo-Reference Electrode

**Figure 5:**
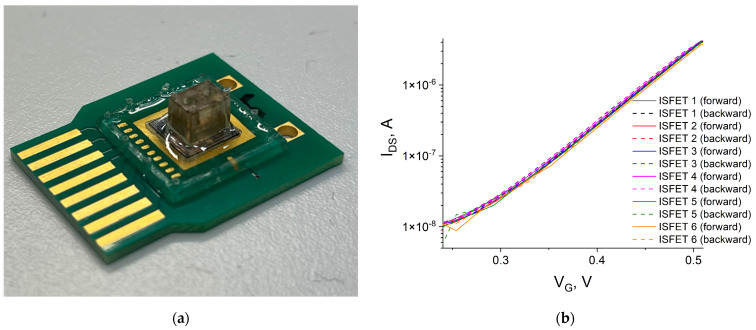
(**a**) Packaged chip; (**b**) I–V curves of the packaged ISFETs.

To detect an enzymatic reaction from blood samples using signal accumulation, it is necessary that the baseline drift would be significantly lower than the signal from the reaction for the selected measurement time. In ISFET structures, baseline drift is related to electrochemical non-equilibrium conditions at the insulator–solution interface due to parasitic capacitance [35,36]. In this work, we used ISFETs specifically designed to minimize the factors impacting parasitic capacitance effects [20]. To test the possibility of carrying out long-term measurements in order to establish the activity of the GLA enzyme at lower concentrations, the sensors were packaged on a printed circuit board and a closed reaction chamber was formed on their surface (Figure 5a). The packaged chip contained six sensors with Au wire as a pseudo-reference electrode, and the signal was simultaneously read from each sensor using the developed portable device. The use of a metal pseudo-reference electrode allows for the integration of the compact electrode directly into the chip assembly process, but its use in an analytical device requires additional research due to potential side reactions at the metal–electrolyte interface [37]. Thus, the reproducibility for the packaged ISFET sensors with Au electrodes was examined. For this, ISFETs were immersed in 20 mM citrate–phosphate buffer with a pH of 4.5, and I–V curves (dependence of drain current I_DS_ on the Au electrode voltage V_G_) were obtained (Figure 5b). The average subthreshold slope was 93.6 ± 1.9 mV/decade, with an average hysteresis of 2.15 ± 0.3 mV. This value did not change, even after a long series of measurements, which experimentally proves the stability of the sensitive surface.

**Figure 6:**
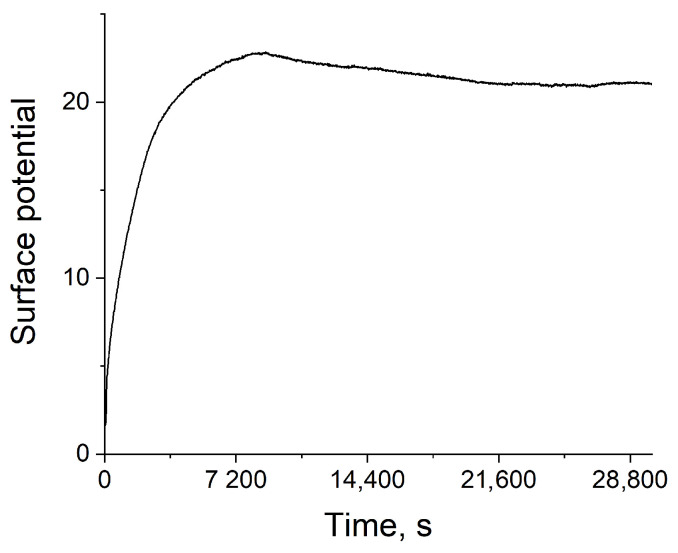
Stabilization of ISFET baseline in 20 mM citrate–phosphate buffer with a pH of 4.5.

The chips were stored dry before measurements. It was experimentally established that potentiometric sensors should be conditioned in buffer solution with an applied voltage before starting the studies of their sensing parameters. This is consistent with previously published data [38]. The baseline reached a plateau after 2.5 h of voltage application using a Au reference electrode (Figure 6). After this, the baseline drift in buffer solution did not exceed −0.011 mV/h. The application of the voltage to the gate generates a difference in potential at the electrolyte–metal interface, which is unique for each electrolyte–metal pair. It is currently believed that the current increase in the ISFET channel is associated with an increase in redox potential due to the redox reaction of the metal surface with dissolved oxygen [37]. This concept is confirmed by long-term experiments, which demonstrate that the ISFET structures are stable for many hours after initial stabilization, if the gate solution does not directly contact the air. These results indicate that sensors with Au electrodes are stabilized after reaching the equilibrium and display highly reproducible sensing characteristics, wherein, after reaching a plateau, the stability of packaged chips with a Au reference electrode is better compared to the results obtained for measurements of chips with a Ag/AgCl electrode (0.092 mV/h) due to absence of evaporation from the well. At the same time, for the Ag/AgCl electrode, stabilization occurred immediately, which allows us to conclude that in the assembled chips, the instability of the system is associated with the gold electrode and not with the hafnium oxide film.

It is worth mentioning that the strategy of the long-term accumulation of the signal produced by low enzyme concentrations implies a relatively long reaction time of about several hours (GLA fluorescent assay takes 20 h for reaction [39]), and such a long time required for system stabilization is mitigated by the faster accumulation of the analytical signal at potentiometric detection.

### 3.5. Real Sample Analysis

**Figure 7:**
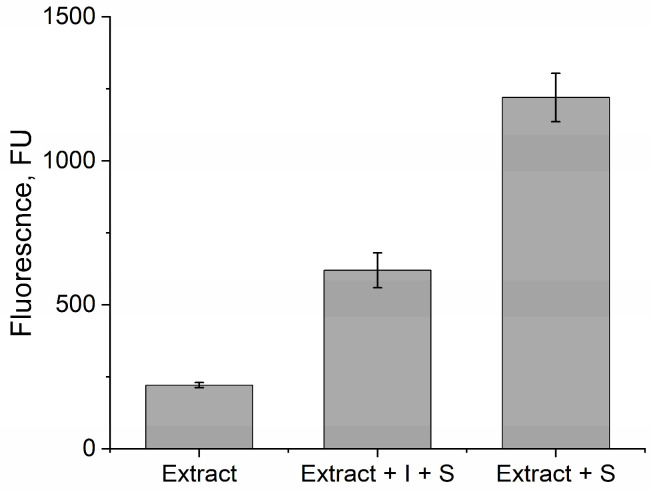
Fluorescence assay of GLA activity in DBS extracts. S—substrate (4-MU-α-Gal), I—inhibitor (deoxygalactonojirimycin).

In newborns, due to the small size and fragility of the fingers, a blood sample is taken from the heel, filling a paper blank with blood. Subsequently, the blood on the blank is stored dry until the sample preparation procedure for a particular analysis is started. The selected 20 mM citrate–phosphate buffer with pH 4.5 was tested for the extraction of GLA from DBSs. The extraction was carried out for 1 h; then, GLA activity was measured in the extracts using a fluorescent assay [39] and the blank (without substrate) and inhibitor controls were set alongside. The addition of GLA inhibitor to the extracts simulated FD, as GLA activity is decreased in people with FD. The fluorescent signal in the test probe was significantly higher compared to both controls (Figure 7). The signal in the inhibitor control sample arose from the activity of α-*N*-acetylgalactosaminidase, which also hydrolyzes 4-MU-α-Gal [40]. Thus, the selected buffer proved to be effective for the extraction of active human GLA.

**Figure 8:**
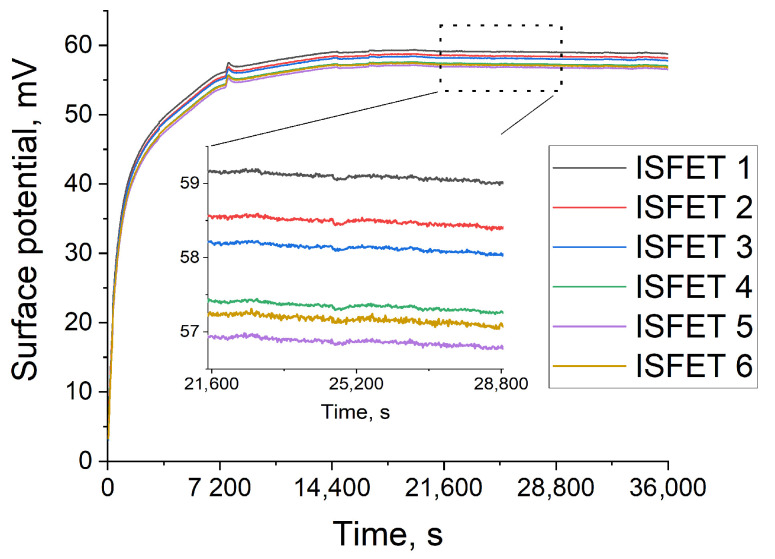
Stabilization of ISFETs baseline with DBS extracts.

The ISFETs’ response upon the addition of the GLA extracts is shown in Figure 8. At the first stage, the potential rapidly increases due to the change in the redox potential of the solution associated with the components extracted from DBSs. However, with further incubation, a stabilization of the signal is observed, with a slight drift of the structures −0.063 mV/h.

**Figure 9:**
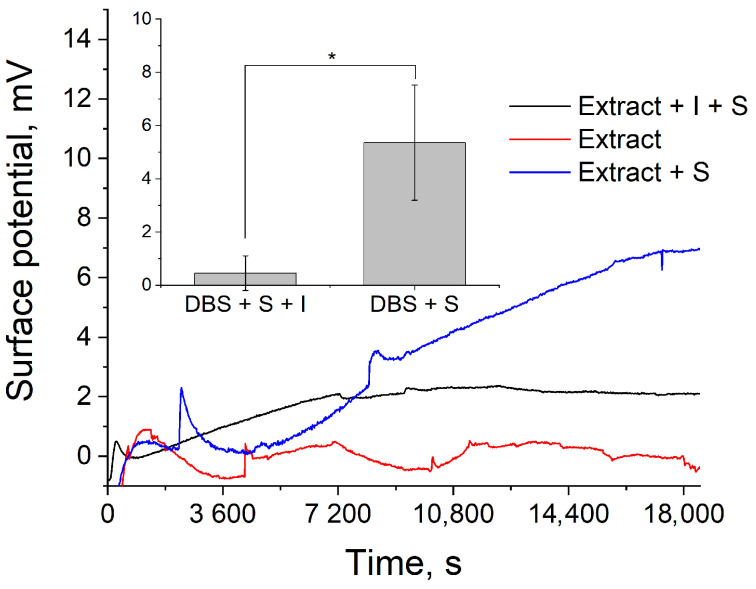
ISFET measurement of GLA activity in DBS extracts. S—substrate (melibiose), I—inhibitor (deoxygalactonojirimycin), *—*p* < 0.05.

The mixtures of DBS extracts with melibiose as substrate and with both melibiose and GLA inhibitor were then analyzed with the ISFET chips (Figure 8). Substrate addition to the DBS extracts launched the catalytic reaction, which resulted in the potential growth in the time interval after sensor stabilization. The addition of GLA inhibitor to the extracts led to the disabling of the reaction, and no obvious potential growth was observed. The difference between mean values of potential increase for DBS extracts with melibiose and with both melibiose and GLA inhibitor was proven to be statistically significant using a *t*-test (*n* = 3, *p* = 0.05) (Figure 9). Thus, ISFET structures allow us to reliably distinguish between the presence and absence of the catalytic activity of an enzyme extracted from DBSs. The analysis time takes 5.5 h, including 2.5 h for signal stabilization and 3 h for reaction detection. In control studies using a fluorescent assay, the result was obtained using incubation for 20 h. Thus, despite the long time taken to establish equilibrium, due to the reaction in a smaller volume with the substrate in saturation concentration (more optimal conditions for catalysis), the absence of the substrate inhibition effect and the signal intensity reduction due to fluorescence quenching in the extracts (see Figure S4), the analysis time turned out to be significantly lower. Reducing the stabilization time is the subject of further design optimization research and can be achieved by developing a method for forming an integrated reference electrode; for example, by using additional treatments or chemical modification of the surface [41]. In addition, the employed ISFET formation technology allows its monolithic integration with circuit solutions for automatic drift compensation; for example, the ISFET/REFET circuit [42], which may make it possible to not have to wait for system stabilization. Further studies are required to determine the cut-off value for GLA activity detected with the developed device.

## 4. Conclusions

In this work, we have shown that ISFETs with Au wire as a reference electrode can be used to investigate the presence of GLA enzyme activity. Despite the fact that the stabilization of the system takes a significant amount of analysis time, after the onset of equilibrium, the developed system allows for the accumulation of a signal from the activity of enzymes extracted from blood samples. To our knowledge, this is the first reported electrochemical method that has been used for the detection of enzyme activity for the purpose of newborn screening. In the future, this will allow for the detection of abnormal enzyme activities in a small-volume sample, enabling the identification of potential patients with a genetic abnormality. The undoubted advantages of the proposed method include the use of direct label-free detection, which can be adapted to measure the activity of various enzymes using native substrates, without changing the hardware component of the analysis.

## Data Availability

Data are contained within the article and Supplementary Materials.

## Supplementary Materials

The following supporting information can be downloaded at: https://www.mdpi.com/article/10.3390/s24113681/s1, Figure S1. TEM image of a hafnium oxide film on top of an aluminum oxide film grown during surface preparation. Figure S2. Time dependence of ISFET response upon addition of 20 nM GLA (Buffer + GLA), 150 μM substrate 4-methylumbelliferyl-α-d-galactopyranoside (Buffer + S) and both of them (Reaction) to 10 mM citrate-phosphate buffer, pH 4.5. Figure S3. Time dependencies of ISFET response to the reaction catalyzed by GLA in 100 mM citrate-phosphate and 100 mM citrate-phosphate buffers, pH 4.5. 1.5 mM 4-methylumbelliferyl-α-d-galactopyranoside was used as substrate. Enzyme concentration was 20 nM. Figure S4. Demonstration of the quenching of fluorescent signal of GLA reaction by dried blood spots extracts.

## Abbreviations

BEOL	back end of line
CMOS	complementary metal-oxide semiconductor
DBSs	dried blood spots
ISFET	ion-sensitive field effect transistor
FD	Fabry disease
GLA	glycohydrolase α-galactosidase A, α-galactosidase A
NBS	newborn screening
4-MU-α-Gal	4-methylumbelliferyl-α-d-galactopyranoside
