# Supplementary material for: Detection of α-Galactosidase A Reaction in Samples Extracted from Dried Blood Spots Using Ion-Sensitive Field Effect Transistors
Source: Sensors (Basel). 2024 Jun 6;24(11):3681. doi: 10.3390/s24113681 (PMC11175248; doi:10.3390/s24113681)
Supplement: Supplementary file 1 [file sensors-24-03681-s001.zip › sensors-2994291-supplementary.pdf]

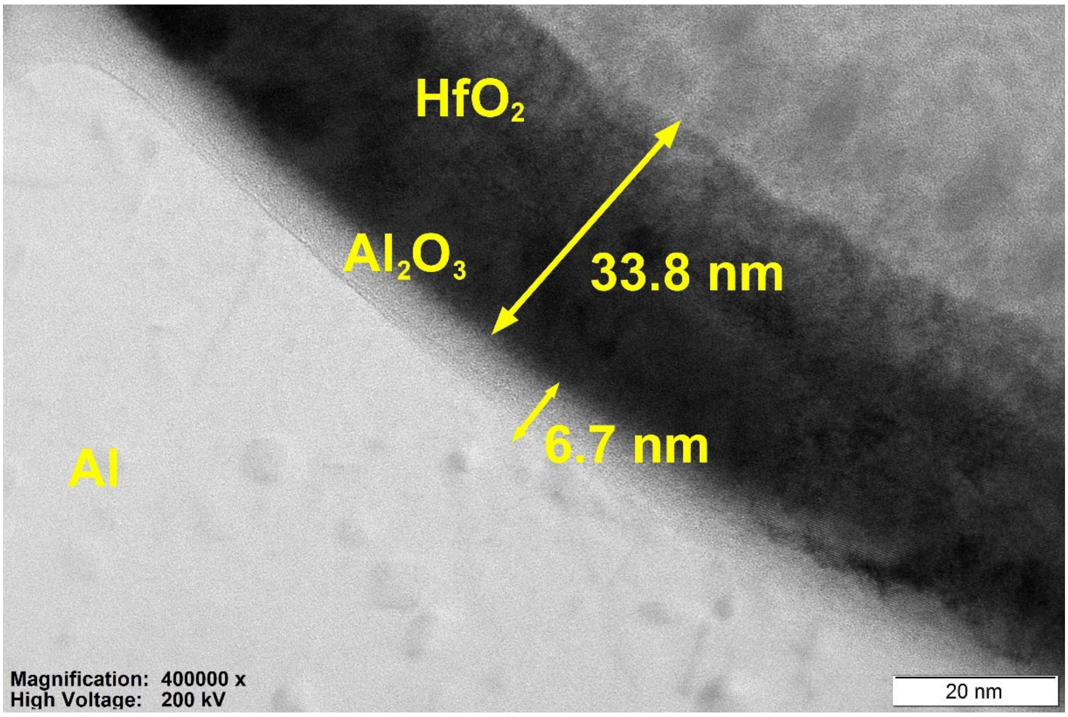

Figure S1. TEM image of a hafnium oxide film on top of an aluminum oxide film grown during surface preparation.

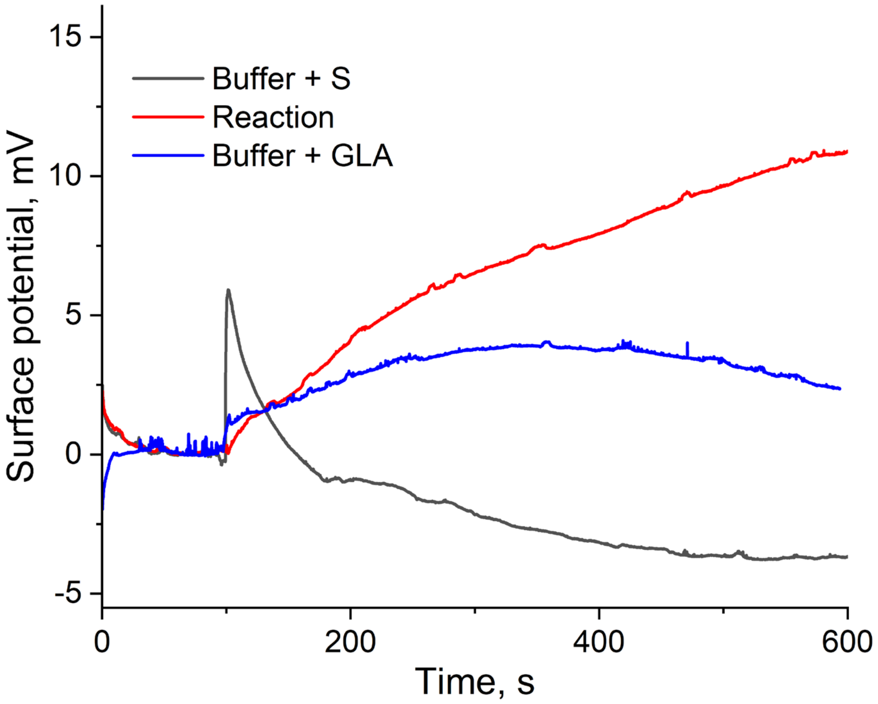

Figure S2. Time dependence of ISFET response upon addition of 20 nM GLA (Buffer + GLA), 150 µM substrate 4-methylumbelliferyl-alpha-D-galactopyranoside (Buffer + S) and both of them (Reaction) to 10 mM citrate-phosphate buffer, pH 4.5.

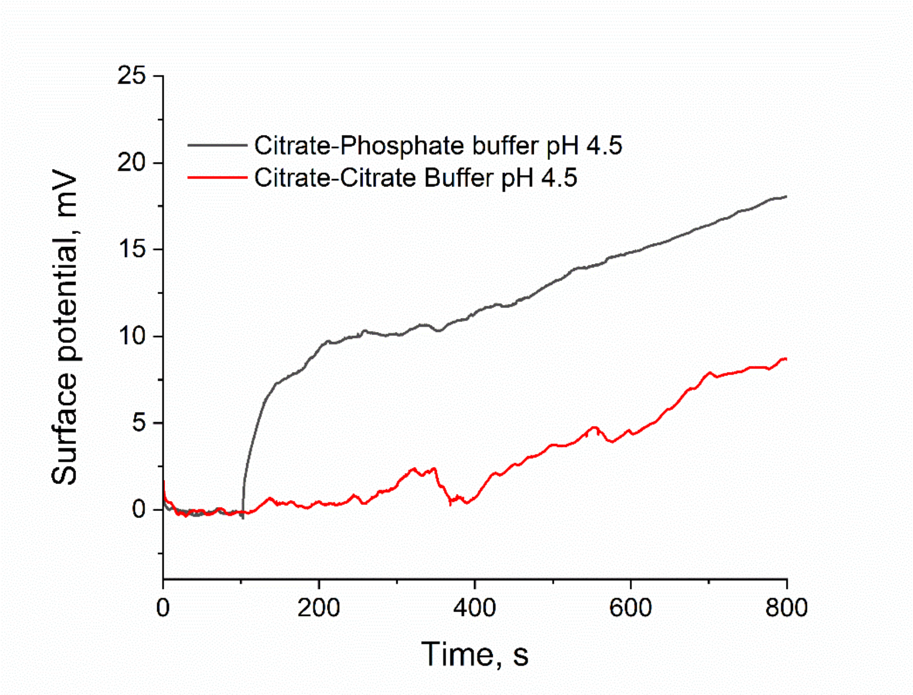

Figure S3. Time dependencies of ISFET response to the reaction catalyzed by GLA in 100 mM citrate-phosphate and 100 mM citrate-phosphate buffers, pH 4.5. 1.5 mM 4-methylumbelliferyl-alpha-D-galactopyranoside was used as substrate. Enzyme concentration was 20 nM.

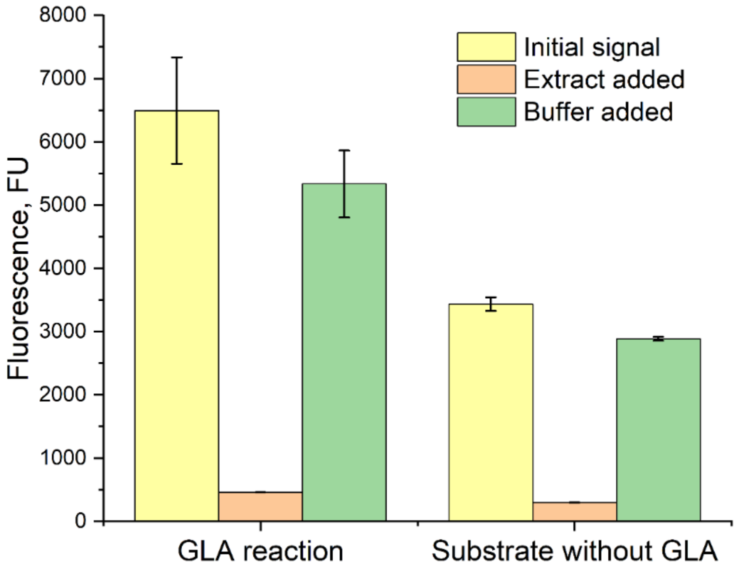

Figure S4. Demonstration of the quenching of fluorescent signal of GLA reaction by dried blood spots extracts. The mix of GLA and 4-methylumbelliferyl-alpha-D-galactopyranoside and the substrate alone, both in 20 mM citrate-phosphate buffer, pH 4.5, were incubated for 20h at 37C and 1200 rpm. Then stop-solution was added, and the fluorescent signal of the reaction product was detected (yellow bars, initial signal). Both probes were then mixed either with DBS extract or 20 mM citrate-phosphate buffer, pH 4.5, and the fluorescence was detected again (orange, extract added and green, buffer added, respectively).
